# The Phage-Encoded *N*-Acetyltransferase Rac Mediates Inactivation of *Pseudomonas aeruginosa* Transcription by Cleavage of the RNA Polymerase Alpha Subunit

**DOI:** 10.3390/v12090976

**Published:** 2020-09-02

**Authors:** Pieter-Jan Ceyssens, Jeroen De Smet, Jeroen Wagemans, Natalia Akulenko, Evgeny Klimuk, Subray Hedge, Marleen Voet, Hanne Hendrix, Jan Paeshuyse, Bart Landuyt, Hua Xu, John Blanchard, Konstantin Severinov, Rob Lavigne

**Affiliations:** 1 Department of Biosystems, KU Leuven, 3000 Leuven, Belgium; Pieter-Jan.Ceyssens@sciensano.be (P.-J.C.); jeroen.desmet@kuleuven.be (J.D.S.); jeroen.wagemans@kuleuven.be (J.W.); marleen.voet@kuleuven.be (M.V.); hanne.hendrix@kuleuven.be (H.H.); jan.paeshuyse@kuleuven.be (J.P.); 2 Institute of Molecular Genetics, Russian Academy of Sciences, 119334 Moscow, Russia; n.akulenko11@gmail.com (N.A.); jonikl@gmail.com (E.K.); severik@waksman.rutgers.edu (K.S.); 3 Department of Biochemistry, Albert Einstein College of Medicine, New York, NY 10461, USA; subray.hegde@einstein.yu.edu (S.H.); Hua.Xu@einstein.yu.edu (H.X.); john.blanchard@einstein.yu.edu (J.B.); 4 Department of Biology, KU Leuven, 3000 Leuven, Belgium; bart.landuyt@kuleuven.be

**Keywords:** phage-induced acetylation, host transcriptional shutdown, phage–host interactions

## Abstract

In this study, we describe the biological function of the phage-encoded protein RNA polymerase alpha subunit cleavage protein (Rac), a predicted Gcn5-related acetyltransferase encoded by phiKMV-like viruses. These phages encode a single-subunit RNA polymerase for transcription of their late (structure- and lysis-associated) genes, whereas the bacterial RNA polymerase is used at the earlier stages of infection. Rac mediates the inactivation of bacterial transcription by introducing a specific cleavage in the α subunit of the bacterial RNA polymerase. This cleavage occurs within the flexible linker sequence and disconnects the C-terminal domain, required for transcription initiation from most highly active cellular promoters. To achieve this, Rac likely taps into a novel post-translational modification (PTM) mechanism within the host *Pseudomonas aeruginosa*. From an evolutionary perspective, this novel phage-encoded regulation mechanism confirms the importance of PTMs in the prokaryotic metabolism and represents a new way by which phages can hijack the bacterial host metabolism.

## 1. Introduction

Bacterial transcription is catalyzed by DNA-dependent RNA polymerase (RNAP). This highly conserved protein complex consists of a catalytically competent core (subunits α_2_ββ’ω) which is capable of RNA synthesis and factor-independent termination. The core enzyme associates with σ subunits, forming holoenzymes, which locate promoters specified by σ subunits and form the transcription initiation complexes [1]. Apart from the σ subunit, the α RNAP subunit also plays a pivotal role in transcription initiation. While the *N*-terminal part of this subunit (α NTD) serves as scaffold for RNAP assembly, the C-terminal domain (α CTD) activates transcription at many promoters by interacting with transcription activators or by binding AT-rich Upstream Promoter (UP) elements [2,3]. A flexible linker of at least 13 amino acids in length connects both domains, although insertions up to 16 amino acids are tolerated in the *Escherichia coli* α RNAP subunits [4].

Most tailed bacteriophages use this transcriptional machinery of bacterial hosts they infect for the transcription of early, late or all viral genes. To achieve this, phages evolved their promoter sequences to resemble bacterial promoters. Some phages change the specificity of the bacterial RNAP by introducing covalent modifications, like the ADP-ribosylation of the *E. coli* α subunit by the ModA, ModB and Alt enzymes of phage T4 [5,6]. Another type of functional hijacking is directed by phage proteins, or so-called HAFs for host acquisition factors, which bind the RNAP at specific stages of infection (reviewed in [7,8]).

Reversible (N^ε^-) lysine acetylation is a post-translational modification (PTM), which was long believed to be rare or nonexistent in prokaryotes. However, global proteomic studies revealed a wealth of acetylated enzymes in *E. coli* [9], *Erwinia amylovora* [10], *Bacillus subtilis* [11], *Salmonella enterica* [12], *Pseudomonas aeruginosa* [13,14] and many other bacteria [15]. Lysine acetylation is a modification that can dramatically change the function of a protein by the neutralization of its charge, which in turn may influence protein structure and interactions with substrates, cofactors and other macromolecules [16]. Acetylomics show that this PTM not only affects metabolic enzymes, but also proteins involved in protein synthesis and turnover, chemotaxis, adaptation, biofilm formation and virulence [15,17,18]. The functional modulation of bacterial proteins through acetylation has also been shown for transcription factors [19,20,21,22]. For example, Kim et al. [22] recently showed that the housekeeping sigma factor HrdB of *Streptomyces venezuelae* is acetylated throughout its growth, thereby enhancing the interaction of HrdB with the RNAP core enzyme as well as the binding activity of the RNAP holoenzyme to target promoters in vivo. However, to date, no example of host takeover directed by a bacteriophage-induced lysine acetylation have been documented.

In this study, we focus on obligatory lytic *P. aeruginosa* phages belonging to a close-knit *Phikmvvirus* genus, part of the *Krylovirinae* subfamily [23,24]. At the onset of their infection, strong σ^70^-like promoters recruit the bacterial RNAP for the transcription of early and middle (DNA replication) genes of the phage. They also encode, directly upstream from the structural gene cluster, a single-subunit RNAP (vRNAP, Gp26) that transcribes structural and lysis-associated genes. Previously, we discovered that the C-terminal domain of the preceding protein (Gp25.1) can bind the β’ RNAP subunit and inactivate bacterial transcription [25], likely controlling the switch to late transcription.

The data presented in this paper demonstrate that some phiKMV-related phages use a second, unique control mechanism to inactivate the bacterial RNAP. Infection by these phages leads to a single proteolytic cleavage within the α RNAP subunit linker. We show that this cleavage is provoked by a phage-encoded acetyltransferase (RNA polymerase alpha subunit cleavage protein or “Rac”), which presumably targets the ClpX and HslU proteases of *P. aeruginosa*. As such, this study describes a novel method of viral transcriptional shutdown and the first example of phage modulation of host RNAP activity using acetylation.

## 2. Materials and Methods

### 2.1. Bacterial Manipulations and Bacteriophages

All *Pseudomonas* strains used were derived from *P. aeruginosa* PAO1 [26] and were grown at 37 °C in lysogeny broth. The *P. aeruginosa rpoA::strep* and *rpoC::protein A* fusion mutants were constructed as described by Vandenbossche et al. [27]. Protease single-gene knockout strains were ordered from the Seattle *P. aeruginosa* PAO1 transposon mutant library [28]. The double knockout *P. aeruginosa* strain Δ*clpX* Δ*hslU* was created by double homologous recombination, mediated by suicide plasmid pME3087 [29]. Briefly, two +/− 700 bp fragments flanking the 5′ and 3′ end of the target gene *hslU* were cloned consecutively in pME3087. Using triparental conjugation, the construct was transferred to *P. aeruginosa* Δ*clpX* [28], leading to two recombination events. All *P. aeruginosa* mutants containing an isopropyl-β-D-thiogalactoside (IPTG)-inducible chromosomal copy of a phage ORF were constructed and grown as described elsewhere [30,31,32].

To mutate the residues of the Rac acetyl-CoA binding site to alanines, two primers flanking the target region were designed, containing the targeted mutations in a 5′ primer tail. After phosphorylation of the primers with T4 polynucleotide kinase (Thermo Scientific, Waltham, MA, USA), the original pUC18-mini-Tn7T-LAC-*rac* vector construct was used as a template in a whole-plasmid PCR reaction using Phusion DNA polymerase (Thermo Scientific). Finally, the PCR product was ligated with T4 DNA ligase (New England Biolabs, Ipswich, MA, USA) to circularize the constructed plasmid. After DNA sequence verification, the mutated construct was integrated in *P. aeruginosa rpoA::strep*, after curation of the FRT-flanked gentamicin resistance marker with Flp recombinase [30].

Bacteriophages were amplified from confluently lysed double-agar layer plates, purified by 0.45-µm filtration and concentrated with polyethylene glycol 8000 as described previously [33].

### 2.2. Protein Expression and Purification

For native RNAP purification during phage infection, a *P. aeruginosa* culture was infected with LUZ19 at a multiplicity of infection of 10. A cell culture (0.5 L) was collected before infection and at 10 and 20 min after infection. The RNAP core enzyme was purified from this cell culture as previously described [34], with some modifications. Briefly, cell pellets were resuspended in buffer A (50 mM Tris-HCl pH 8.0, 10 mM ethylenediaminetetraacetic acid (EDTA), 5% (*v*/*v*) glycerol, 1 mM dithiothreitol (DTT), 300 mM NaCl, 0.3 mg/mL lysozyme) and incubated for 20 min for lysozyme digestion. The cells were then lysed by sonication, after which the lysate was centrifuged at 8000× *g* for 30 min. A 10% solution of Polymin P at pH 7.9, was slowly added to the supernatant with stirring, to a final concentration of 0.8%. Stirring was continued for an additional 10 min, followed by centrifugation at 12,000× *g* for 15 min. The pellet was thoroughly resuspended in TGED buffer (10 mM Tris-HCl pH 8.0, 5% (*v*/*v*) glycerol, 0.5 mM EDTA, 0.1 mM DTT) plus 0.5 M NaCl with the aid of a glass rod. The suspension was centrifuged, and the supernatant was discarded. The pellet washing cycle was repeated at least five times until no protein was detectable in the supernatant. To elute RNAP from Polymin P, the pellet was resuspended in TGED plus 1 M NaCl. The mixture was centrifuged at 12,000× *g* for 30 min. Finally, ground ammonium sulfate was slowly added to the supernatant with stirring to the amount of 0.35 g per 1 mL solution. The pH was adjusted to pH 7.0–7.5 with 2 N NaOH, and the mixture was incubated overnight. Ammonium sulfate suspension of the Polymin P eluate was centrifuged, and the pellet was resuspended in 100-fold volume of buffer TGED and applied on a 1 mL HiTrap Heparin HP column (Cytiva, Marlborough, MA, USA), equilibrated with TGED. The column was washed with 10 column volumes of TGED containing 0.3 M NaCl, and native RNAP was eluted with 5 mL of TGED containing 0.6 M NaCl. Pooled column fractions were concentrated to 0.5 mL in Amicon devices with a 100 K cutoff, diluted tenfold using storage buffer (40 mM Tris-HCl pH 7.9, 0.2 M KCl, 50% (*v*/*v*) glycerol, 1 mM EDTA, 1 mM DTT), concentrated to approximately 1 mg/mL and stored at −20 °C. The recombinant σ^70^ subunit was purified according to Tang et al. [35].

Cultures (600 mL) of the *P. aeruginosa rpoA::strep* and *rpoC::protein A* fusion mutants were infected with phage as described previously [27]. The *P. aeruginosa rpoA::strep* mutants containing an inducible LUZ19 gene in their genome were grown in 500-mL cultures until they reached an optical density (OD) at 600 nm of 0.1. Next, LUZ19 protein expression was induced by adding 1 mM IPTG and incubation at 37 °C for 30 min.

After phage infection or phage protein induction, cell growth was immediately halted by incubation of the culture in icy water for 8 min (while shaking). Cells were pelleted (4000× *g*, 45 min, 4 °C) and lysed as described previously [27]. *P. aeruginosa* RpoA-Strep proteins were purified by loading the obtained soluble protein fraction on a Bio-Rad (Hercules, CA, USA) Poly-Prep Chromatography column containing 1 mL pre-washed Strep-Tactin^®^ Sepharose beads (IBA Lifesciences, Goettingen, Germany). The beads were washed five times with 1 mL wash buffer (100 mM Tris HCl pH 8.0, 150 nM NaCl, 1 mM EDTA) and eluted five times with 1 mL elution buffer (wash buffer + 2.5 mM HABA). The eluted fractions were pooled and concentrated by ultrafiltration (Amicon Ultra 3K, Merck Millipore, Darmstadt, Germany). RpoC–Protein A fusion proteins were purified using IgG SepharoseTM 6 Fast Flow beads (Cytiva) and eluted by cleavage by the proTEV Protease (Promega, Madison, WI, USA), as described previously [25].

The Rac coding sequence was PCR amplified using phage DNA as template and cloned into the Novagen pET29a vector (Merck Millipore) as an NdeI/HindIII product. Recombinant Rac bearing a C-terminal His_6_ tag was produced in *E. coli* T7 Express (New England Biolabs), harboring the plasmid pGroESL-911 that expresses the molecular chaperone GroES/GroEL. In total, 1 L of lysogeny broth supplemented with kanamycin (35 µg/mL) and tetracycline (8 µg/mL) was inoculated with 10 mL of an overnight culture and incubated at 37 °C. The culture was grown to mid-log phase (OD_600_ of 0.6), cooled to 20 °C, induced with 0.5 mM IPTG, and further incubated overnight at 20 °C. Recombinant Rac was purified using Ni-NTA chromatography (Cytiva). Protein concentrations were estimated by the Bio-Rad protein assay method using bovine serum albumin (BSA) as a standard.

### 2.3. Mass Spectrometry Identifications

To determine the mass of the α CTD fragment, desalted peptides (ZipTip C18, Merck Millipore) of this domain were dissolved in 0.1% trifluoroacetic acid (TFA) in 60% acetonitril and subjected to Fourier transform ion cyclotron resonance (FTICR) mass spectrometry on an APEX-Qe, equipped with a 9.4 Tesla magnet (Bruker Daltonics, Bremen, Germany). Spectra were analyzed in DataAnalysis 4.0 (Bruker Daltonics).

Alternatively, accurate mass measurements of the subunit fragments were performed with MALDI-TOF/TOF. First, the protein spots were excised from the gel and digested with trypsin. Briefly, gel pieces (1–2 mm^3^) were washed to remove dye, dehydrated with acetonitrile (ACN), and rehydrated with 4 µL of digestion solution containing 20 mM ammonium bicarbonate and 15 ng/µL sequencing-grade trypsin (Promega). The tryptic digestion was incubated at 37 °C overnight, after which the peptides were extracted from the gel with 10 µL of 10% ACN containing 0.5% TFA. In total, 2 µL of each extract was mixed with 0.5 µL 2,5-dihydroxybenzoic acid saturated solution in 20% ACN containing 0.5% TFA on the stainless steel MALDI sample target plate and dried. Mass spectra were recorded on an Ultraflex II MALDI-TOF/TOF mass spectrometer (Bruker Daltonics) equipped with Nd laser (354 nm). The MH+ molecular ions were detected in reflection mode; the accuracy of monoisotopic mass peak measurement was 70 ppm. Spectra were analyzed using the Mascot software (Matrix Science, London, UK) through the NCBI database. Partial oxidation of methionine residues and propionamidomethylation of cysteine was permitted; up to one missed tryptic cleavage was considered for all tryptic mass searches. Protein scores greater than 87 were considered as significant (*p* < 0.05).

Routine peptide identification in SDS-PAGE protein bands was performed by in-gel trypsinization followed by LC-MS/MS identification on an Easy-nLC 1000 liquid chromatograph (Thermo Scientific) that was online-coupled to a mass calibrated LTQ-Orbitrap Velos Pro (Thermo Scientific) as described previously [36]. The analysis of the mass spectrometric raw data was carried out using Proteome Discoverer software v.1.3 (Thermo Scientific).

### 2.4. Pulse Labeling with ^3^H-Uridine

The impact of Rac expression on *P. aeruginosa* PAO1 transcription was investigated using tritium-labelled uridine precursors, as described previously [31]. Briefly, an exponentially growing culture (OD_600_ of 0.3) was labeled for 10 min with 1 μCi/mL (5,6-^3^H)-uridine (PerkinElmer, Waltham, MA, USA). Samples were taken 0, 10, 30 and 60 min after the induction of Rac with 1 mM IPTG and precipitated in 5% ice-cold trichloroacetic acid (TCA). The precipitate was transferred to a Unifilter-96 GF/C (PerkinElmer) using a Filtermate 96 Harvester (Packard) and washed. After the addition of MicroScint 0 (PerkinElmer), the radioactive signal was measured with a TopCount NXT Microplate Scintillation Counter (PerkinElmer). The experiment was repeated on three independent occasions. Non-induced cultures were grown in parallel to permit normalization for each timepoint.

### 2.5. In Vitro Transcription

The in vitro transcriptional activity of purified RNAP complexes was assayed by adding 20 nM promoter DNA, 100 μM each of ATP, CTP, and GTP, 10 μM UTP and 0.4 µCi of (α-^32^P) UTP in buffer R (40 mM Tris-HCl, 40 mM KCl, 10 mM MgCl_2_, 5 mM DTT, 100 µg/mL BSA) to a 100-nM core enzyme purified from infected cells in a 10-µL reaction volume. Reactions were allowed to proceed for 10 min at 37 °C with and without the presence of 200 nM recombinant σ^70^ and were terminated by the addition of an equal volume of denaturing loading buffer. The resulting products were resolved on a denaturing 6 M urea 20% (*w*/*v*) polyacrylamide gels and visualized using a Typhoon PhosphorImager (Cytiva).

### 2.6. Western Immunoblot Analyses

Cells were sonicated on ice for 3–5 min with 5 s pulses (40% intensity) and 5 s intervals. Soluble proteins were collected by centrifugation (30 min, 18,000× *g*, 4 °C), concentrated by acetone precipitation, loaded on a 12% SDS-PAGE gel (150 µg/sample), transferred to a polyvinylidene fluoride (PVDF) membrane and subjected to Western immunoblot analysis. To detect acetylated lysine residues, we applied a cocktail of two polyclonal anti-acetyl lysine antibodies (Cell Signaling Technologies, Leiden, The Netherlands and ImmuneChem Pharmaceuticals, Burnaby, BC, Canada). Alternatively, for the detection of the intact α RNAP subunit, we used the RNA Polymerase Alpha Monoclonal Antibody (Neoclone^®^, BioLegend, San Diego, CA, USA), which recognizes epitope 209–329, covering both the α NTD, CTD and the linker domain. After washing, secondary horse radish peroxidase conjugated goat anti-rabbit IgG (Cell Signaling Technologies) or goat anti-mouse IgG (BioLegend) antibodies were applied for one hour at room temperature. Enhanced chemiluminescence reagents (Cell Signaling Technologies) were used for visualization.

### 2.7. Acetyltransferase Substrate Profiling

Freshly prepared *P. aeruginosa* cell lysate (from mid-log phase culture) containing 1 mg/mL total proteins was incubated with 5 μM purified recombinant Rac and 160 μM acetyl-CoA in 5 mL acetylation buffer (50 mM HEPES, 100 mM NaCl, pH 7.5) at 37 °C for two hours. Rac-His_6_ was captured by 0.5 mL Ni-NTA agarose and removed by centrifugation at 4 °C. The supernatant was incubated with 250 µL anti-acetyl lysine agarose (ImmuneChem Pharmaceuticals) pre-washed with 3 mL ice cold 50 mM MOPS buffer (pH 7.5). After overnight incubation at 4 °C, the agarose was washed with 1 mL NETN buffer (0.5% NP40, 1 mM EDTA, 50 mM Tris, 100 mM NaCl, pH 8.0), 3 mL ETN buffer (1 mM EDTA, 50 mM Tris, 100 mM NaCl, pH 8.0) and 1 mL of 50-mM 3-(*N*-morpholino)propanesulfonic acid (MOPS) buffer (pH 7.5) successively. The bound proteins were eluted by 1 mL elution buffer (8 M urea, 100 mM NaH_2_PO_4_, 10 mM Tris, pH 8.2). The eluates were concentrated by ultrafiltration using an Amicon 3K cutoff membrane and subjected to SDS-PAGE. Cell lysates treated identically in the absence of Rac served as controls.

## 3. Results

### 3.1. Bacteriophage LUZ19 Infection Leads to A Cleavage of the Host RNAP α Subunit

**Figure 1:**
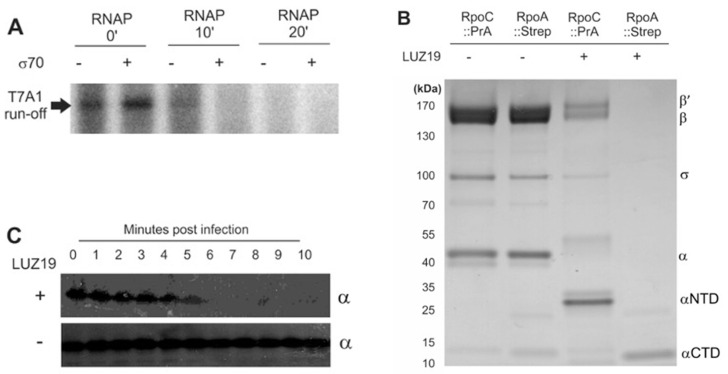
The bacterial DNA-dependent RNA polymerase (RNAP) complex is cleaved and inactivated during phage infection. (**A**) In vitro transcription by the *P. aeruginosa* RNAP complex isolated before and during LUZ19 infection. (**B**) Pull-down analyses of the *P. aeruginosa* RNAP complex after 10 min of phage infection. Purification were mediated by Protein A or Strep affinity tags fused to the C-terminus of the β’ (RpoC) and α (RpoA) subunits. Eluates were separated on a 4–15% SDS-PAGE gel. (**C**) Western blot analysis of bacterial cell lysates collected at indicated times post infection. We collected phage-infected bacterial cells with single-minute intervals between one and ten minutes post infection, and compared to the uninfected sample (time point 0). Equal amounts of the soluble protein fraction of these samples were subjected to Western Blot using an antibody specific for the α RNAP subunit. N/C-terminal domain (NTD, CTD).

The central question of this research stems from an observation made during a proteome analysis of the infection process by *Pseudomonas* phage LUZ19 (a *Phikmvvirus* phage) of its *P. aeruginosa* PAO1 host. Two-dimensional gel analysis revealed an unexpected shift in the protein mass of the host RNAP α subunit, observed 10 min post infection [37]. Using a promoter-specific transcription initiation assay, we observed that RNAP isolated from infected cells was unable to initiate transcription from a strong σ^70^-dependent T7 A1 promoter (Figure 1A). This shows that LUZ19 inactivates bacterial transcription in the course of infection.

To analyze the bacterial RNAP under viral attack in more detail, we constructed *P. aeruginosa* PAO1 strains carrying Protein A and Strep II^TM^ affinity tags fused to the C-termini of the β’ and α RNAP subunits, respectively [27]. The mutant strains were viable and indistinguishable from the parental wildtype strains under laboratory and phage infection conditions. After 10 min of infection by LUZ19, the host RNAP complex was purified, separated and visualized on SDS-PAGE (Figure 1B). With tagged β’, the RNAP complex was affinity purified, but a band at the expected height for the α subunit was absent. Instead, another intense band was apparent, about 10 kDa shorter in mass. A similar pull-down experiment using the C-terminal *rpoA* fusion yielded a single protein of approximately 12 kDa in mass, but the other components of the RNAP complex were missing. MALDI-MS analysis demonstrated that the shortened version of α in material pulled down through RNAP β’-contained peptides from the α *N*-terminal domain (NTD), while the 12 kDa band detected in the α pull-down contained peptides from the α C-terminal domain (CTD).

**Figure A1:**
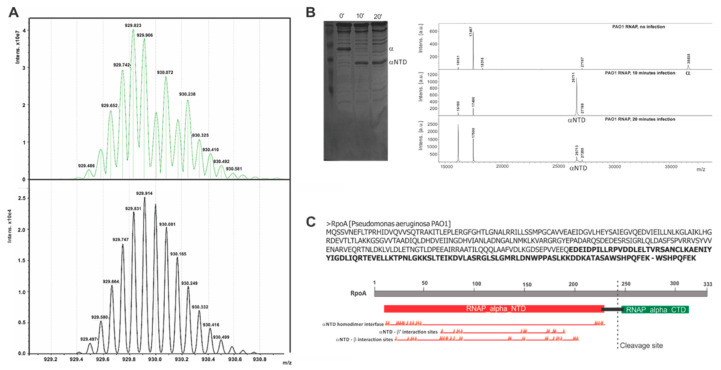
Determination of cleavage site within C-terminal domain (α CTD). (**A**) The cleaved α CTD was purified from phage-infected cells using the C-terminally fused Strep II^TM^-tag (WSHPQFEK) and subjected to Fourier mass spectrometry (APEX-Qe, Bruker Daltonics). The protein mass (including the tag) was calculated from the spectrum (in green) to be 11,157.87 Da, corresponding to the RpoA fragment indicated in bold in the primary sequence. The bottom figure (in black) represents the theoretical spectrum of this α CTD fragment, with a predicted mass of 11,152.96 Da. The cleavage site is located in the flexible linker sequence connecting the two functional domains. (**B**) Alternative determination of cleavage site within the α CTD domain. The entire RNAP complex was purified from phage-infected cells, separated on SDS-PAGE (left panel) and subjected to MALDI-TOF mass spectrometry (right panel). The relevant m/z range is shown. After ten minutes of infection, we noticed the disappearance of the peak corresponding to the original α subunit (38,835 Da), while a single alternative peak at 26,712 ± 1 Da appeared. This mass allowed us to determine the cleavage site within the flexible linker. (**C**) The different domains of RpoA are shown with the exact position of the cleavage site.

To determine the exact cleavage site within the α RNAP subunit, the Strep II^TM^-tagged C-terminal domain of the α RNAP subunit (α CTD) (Figure 1B, lane 4) was analyzed using Fourier transformation (FT)-MS. This analysis allowed us to exactly determine the mass of this fragment to be 11,157 Da, corresponding to the stretch of 89 amino acids at the C-terminus of RpoA with a theoretically predicted mass of 11,153 Da (Appendix A, Figure A1A). A MALDI-TOF/TOF analysis performed on the entire RNAP complex used in the transcription assay confirmed this cleavage site, as a decrease in the mass of the α subunit from 38,835 to 26,712 ± 1 Da was observed, which corresponds to the 244 *N*-terminal amino acids of RpoA (α NTD). Moreover, no secondary cleavage products are formed, since only a single peak in the spectrum can be associated with the α NTD (Appendix A, Figure A1B). As such, the Gln^244^-Glu^245^ amide bond, located within the flexible linker domain, which connects the two functional domains of the protein (Appendix A, Figure A1C), was identified as the α RNAP subunit cleavage site. Our analyses show that the entire α CTD is removed from the RNAP complex in the final stage of phage infection, implying the inability of this complex to bind UP elements [2] or bacterial transcriptional activators which function through α CTD binding.

Next, we collected phage-infected bacterial cells with single-minute intervals to determine the timing of this proteolytic processing. Lysates of these samples were subjected to Western Blot using an antibody specific for the intact α RNAP subunit. We observed that the signal disappeared after five to six minutes of phage infection (Figure 1C).

### 3.2. Gene Product 28 Provokes the RNAP Alpha Subunit Cleavage (Rac)

Intriguingly, bioinformatic analysis fails to predict a proteolytic protein or proteolytic domains among the products of *Phikmvvirus* genes. This suggests an indirect effect and/or the involvement of bacterial proteins in the observed cleavage of RNAP α in infected cells. If a single-phage gene were responsible for the α subunit cleavage, expression of this gene would be sufficient to inhibit *P. aeruginosa* growth since the loss of the α CTD is lethal for the host [38]. We therefore performed a screen by cloning the first ten annotated unknown early phage genes and the two genes directly up- and downstream of the viral RNAP in the expression vector pUC18-mini-Tn7T-Lac. Next, we did a transformation of all constructs to the *P. aeruginosa rpoA::strep* mutant. This resulted in a stable integration of the expression cassettes in the *P. aeruginosa rpoA::strep* genome, allowing controlled, single-copy expression of each phage gene [30,31,32].

**Figure 2:**
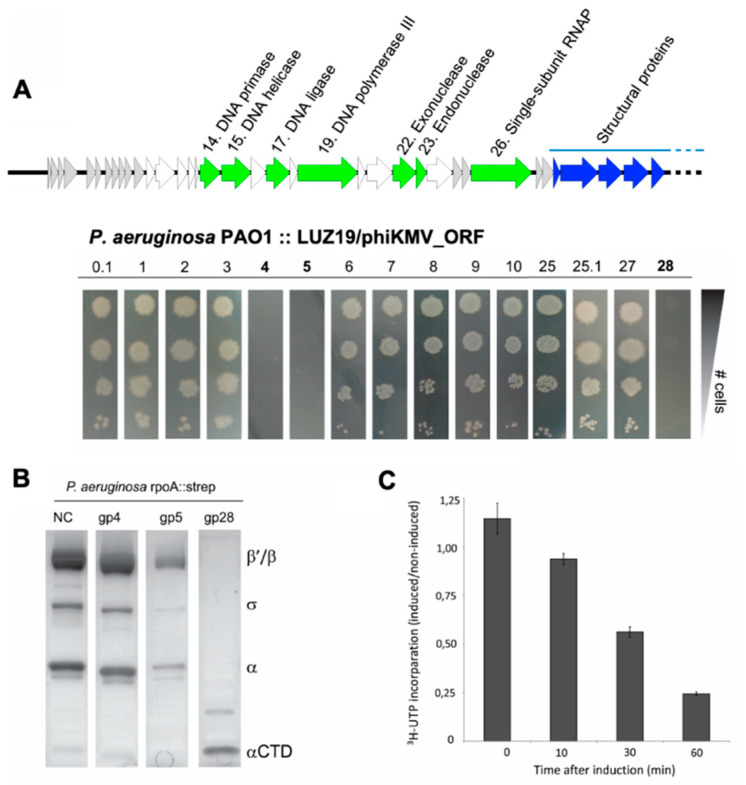
Phage gene 28 is responsible for α subunit cleavage. (**A**) Overview of the first part of the LUZ19 genome. Genes encoding proteins with predicted function are labeled in green, genes encoding structural proteins are blue. The first ten early genes and the two genes up- and downstream of the viral RNAP (highlighted in light grey) were selected and cloned into a single-copy plasmid, integrated in the *P. aeruginosa* PAO1 *rpoA::strep* genome. Dilution series of the cells were plated on LB with 1 mM isopropyl-β-d-thiogalactoside (IPTG) and checked for growth. (**B**) Pull-down analyses of the α RNAP subunit from cells grown in the presence of the indicated phage proteins (after 30 min induction with 1 mM IPTG). As negative control (NC), *P. aeruginosa* PAO1 *rpoA::strep* was used. Gp28 or Rac shows a consistent degradation of the RNAP α subunit. (**C**) Exponentially growing *P. aeruginosa* PAO1 cells containing *Rac* were pulse labeled with ^3^H-uridine in the presence or absence of 1 mM IPTG. Each data point represents the average of three independent samples with standard deviation. For each timepoint, the scintillation counts for the induced sample were normalized to the non-induced sample at the same timepoint.

**Figure A2:**
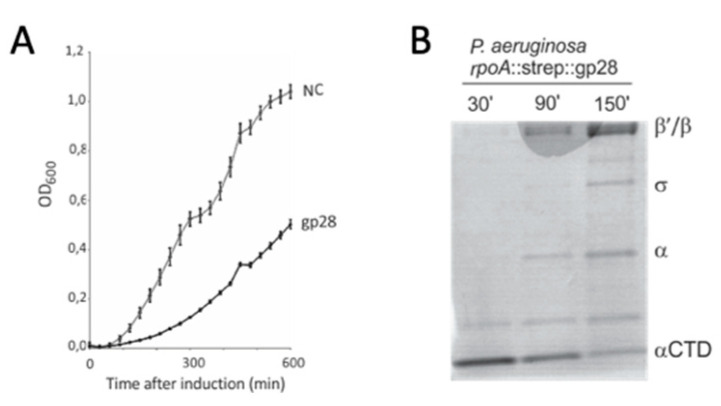
Reversion of the cleaved phenotype upon prolonged incubation. (**A**) Wildtype *P. aeruginosa* PAO1 *rpoA::strep* (NC) and *P. aeruginosa* PAO1 *rpoA::strep,gp28* cells were grown overnight, diluted (1:50) in LB/1 mM IPTG, and growth was followed spectrometrically. (**B**) Pull-down of the α RNAP subunit from 200 mL of *P. aeruginosa* PAO1 *rpoA::strep,gp28* cells after 30, 90 and 150 min of incubation. Eluates were separated on 4–15% SDS-PAGE gels.

Expression of three out of fifteen phage genes tested was found to be detrimental for *P. aeruginosa* growth (Figure 2A). Next, we induced expression of these three genes in large cultures and processed them for RNAP purification by Strep II^TM^ tag affinity chromatography. While the entire RNAP holoenzyme was still present upon induction of Gp4 and Gp5, only the α CTD was purified in the case of Gp28 induction (Figure 2B). This experiment not only shows that LUZ19 Gp28 is the determinant for α CTD cleavage, but also implies that no other phage-encoded factors are required for cleavage. We therefore termed this gene product as Rac, the RNA polymerase alpha subunit cleavage protein. The net effect of Rac on *P. aeruginosa* transcription was determined by measuring 5,6-^3^H-uridine incorporation, showing a decline of 76% after one hour of induction in comparison to the non-induced control cells (Figure 2C). Notably, after prolonged incubation, we observed a reversion of the phenotype and a regrowth of the bacterial cells (Appendix A, Figure A2), though this effect was not investigated further.

### 3.3. The RNAP α Subunit Cleavage Is Triggered by a Predicted Acetyltransferase Encoded by Many phiKMV-Related Viruses

**Figure A3:**
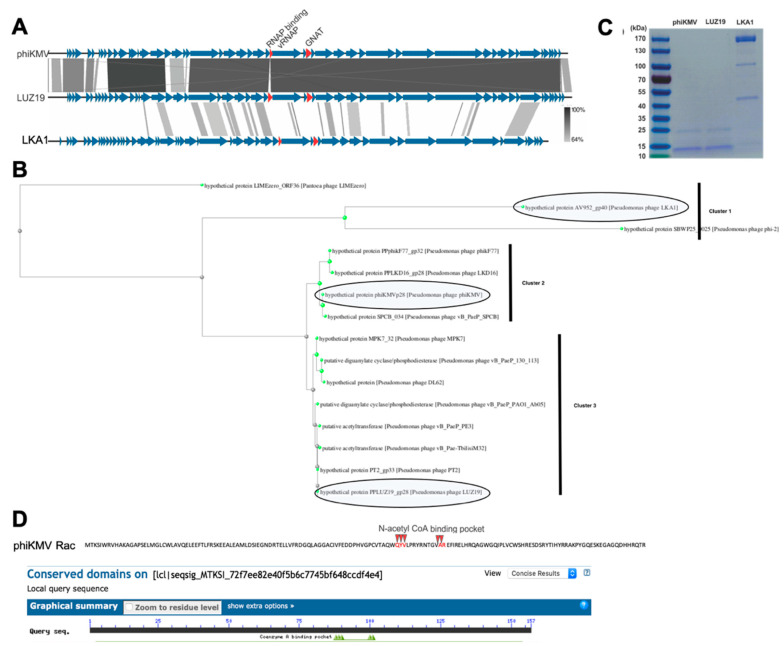
Conservation of Rac among members of *Krylovirinae*. (**A**) Genome comparison of phage phiKMV, LUZ19 and LKA1, scored according to tBLASTx comparison. Genes 25.1 and 28 are indicated with red arrows. (**B**) Phylogenetic tree (neighbor joining). (**C**) Affinity pull-down of *P. aeruginosa rpoA::strep* cells infected with phiKMV, LUZ19 and LKA1, followed by pull-down of the RNAP complex. After separation of these complexes on 12% SDS-PAGE gels, it is clear that the cleaved α RNAP phenotype can only be associated with infection of phages from the clusters with phiKMV and LUZ19. (**D**) Sequence alignment of a selection of Rac-like proteins of various clusters, with indication of the *N*-acetyl CoA binding pocket (QYV---------AR).

The *Rac* gene (encoding Gp28) is located downstream of the phage-encoded RNAP (Gp26) gene, and its coding sequence partly overlaps the first structural gene of the phage. The protein is conserved in the *Phikmvvirus* genus and some other members of the *Krylovirinae* as revealed by a BLASTp analysis (Appendix A, Table A1). Phylogenetic analysis of the primary Rac-like sequences of *Krylovirinae* phages shows division into basically three clusters centered around *Phikmvvirus* members phiKMV and LUZ19 and *Stubburvirus* LKA1, with the latter cluster showing less similarity to LUZ19 Rac (65–73% query coverage, 31.78–37.39% identity) (Appendix A, Figure A3, Table A1). Intriguingly, purification of host RNAP from cells infected by these three phages showed that only *Phikmvvirus* members provoke the RNAP α subunit cleavage (Appendix A, Figure A3), making it a genus-specific mechanism. Despite the presence of a Gcn5-related acetyltransferase (GNAT) homolog gene in a corresponding genetic location, phage LKA1 leaves the α subunit intact during infection.

**Figure A4:**
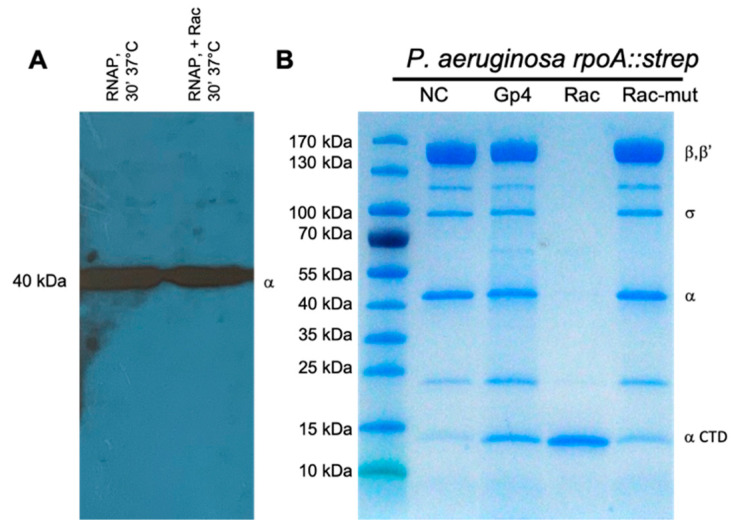
Rac does not have protease activity and the predicted *N*-acetyl CoA binding pocket of Rac is required for cleavage. (**A**) Recombinant Rac was incubated at 37 °C for 30 min with *P. aeruginosa* RNAP purified using the RpoA-Strep tag. Western blot using antibody specific for the intact α RNAP subunit shows the α subunit is not cleaved. (**B**) The RpoA-Strep was purified from *P. aeruginosa* cultures 30 min after induction of Gp4 (control), wildtype Rac or mutated Rac (with an inactivated acetyl-CoA binding pocket). The wildtype *rpoA::strep* mutant was used as a negative control. After RpoA-Strep purification and separation on a 4–20% SDS-PAGE gel, it is clear that only the wildtype Rac provokes the α subunit cleavage.

In vitro incubation of recombinant Rac with RNAP in a protease buffer did not result in cleavage of the RNAP α subunit, excluding direct protease activity of Rac (Appendix A, Figure A4). In silico analysis, however, indicated the clear relatedness of Rac with the family of the Gcn5-related acetyltransferases (GNAT, Appendix A, Table A2), including the five residues comprising the acetyl-CoA binding pocket. To investigate whether this predicted binding pocket is involved in the observed phenotype, its Q, Y, V and R residues were mutated to alanines (Appendix A, Figure A3). RpoA-Strep purification after expression of this mutated Rac restores the wildtype phenotype, indeed suggesting that acetyltransferase activity is required for α subunit cleavage (Appendix A, Figure A4).

The acetylated protein profile in *P. aeruginosa* upon expression of wild-type Rac.

**Figure A5:**
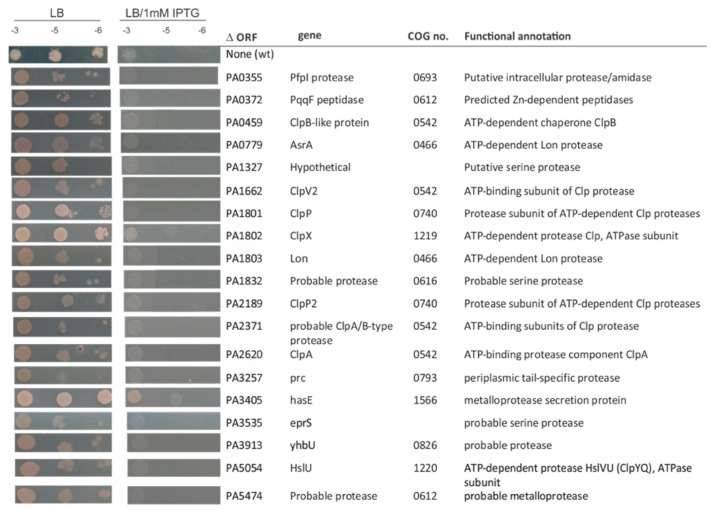
Analysis of Rac induction in Tn-mediated single-gene knockouts of *P. aeruginosa* PAO1. All predicted cytoplasmic proteases in the genome of *P. aeruginosa* were selected and transformed with the pUC18mini-*Rac* construct, which allows stable, single-copy expression of the phage Gcn5-related acetyltransferase (GNAT). The resulting strains were plated on media with and without the presence of 1 mM IPTG, and checked for overnight growth in comparison to the wildtype strain.

As the RNAP cleavage requires Rac, a predicted GNAT that does not contain any predicted protease domains or does not have any protease activity on host RNAP, we hypothesized that Rac acetylates a host protease to change its target specificity. This change in substrate specificity has to be very specific, since no other protein degradation products were detected during the two-dimensional gel electrophoresis analysis of LUZ19-infected cells [37]. *P. aeruginosa* PAO1 encodes at least 26 different proteases, many of which are secreted and act as virulence factors [39]. Therefore, we selected the 19 annotated cytoplasmic proteases [40] and induced expression of Rac in knockout strains of each of these. However, in none of the cases was the reversal of the phenotype observed (Appendix A, Figure A5).

**Figure 3:**
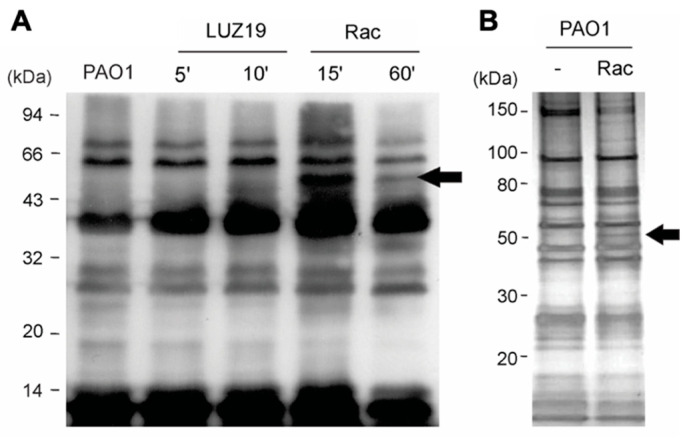
Impact of LUZ19 infection and Rac expression on *P. aeruginosa* PAO1 acetylation. (**A**) Variation in the *P. aeruginosa* acetylome upon infection of LUZ19 and induction of Rac expression. In each lane, 150 µg of cytoplasmic soluble proteins were separated by SDS-PAGE, transferred to a PVDF membrane and subjected to anti-acetyl lysine Western blot. (**B**) SDS-PAGE gel showing the result of an immunoaffinity enrichment of the Rac substrates from *P. aeruginosa* cell lysate. The arrow indicates the additional acetylated protein in the presence of recombinant Rac and acetyl-CoA in comparison to the control sample.

Next, we checked the overall acetylation levels in cellular lysates using Western immunoblot analysis with a cocktail of two polyclonal anti-acetylated lysine antibodies. In phage-infected cells 10 min post infection, we noticed a general increase in the intensity of acetylated bands (Figure 3A, lane 3). When lysates from cells which chromosomally expressed Rac were blotted, we also observed a larger general increase in the acetylation of the *P. aeruginosa* proteins and the appearance of an additional band with an approximate mass of 50 kDa (Figure 3A, lanes 4 and 5). Analogously to the resumption of cell growth observed at later times of Rac induction, the acetylation level of the bacterial lysate decreased after prolonged induction of Rac.

**Figure A6:**
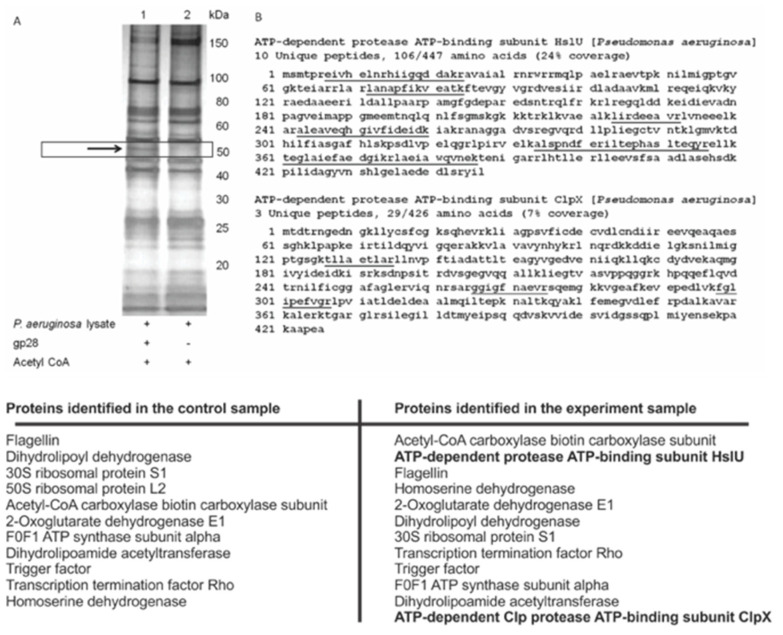
Rac substrate profiling. (**A**) Immunoaffinity enrichment of the Rac substrates from *P. aeruginosa* cell lysate. The arrow indicates the additional acetylated protein in the presence of recombinant Rac and acetyl-CoA (lane 1), compared to the control sample (lane 2). (**B**) MS analysis reveals that HslU and ClpX are present in lane 1 but not in lane 2, with sequence coverage of 24% and 7%, respectively. The identified peptide sequences are underlined.

To explore this result in an in vitro setting, recombinant Rac carrying a C-terminal His_6_ tag was produced in *E. coli* and purified using Ni-NTA. The protein had to be used immediately for downstream assays as it degraded rapidly. For substrate profiling, Rac-His_6_ was mixed with acetyl-CoA and a lysate from *P. aeruginosa* PAO1 cells, and this mixture was subjected to immunoprecipitation using the anti-acetyl lysine antibodies. Upon separation of the bound and eluted proteins by SDS-PAGE, we again noticed the appearance of an additional band around 50 kDa, which was absent in the control sample lacking Rac (Figure 3B). This band and its corresponding position in the control lane were excised and subjected to MS analysis, and were found to contain unique peptides of the *P. aeruginosa* AAA+ ATPases ClpX and HslU, respectively (Appendix A, Figure A6). However, none of the identified peptides contained an acetylated lysine residue.

**Figure A7:**
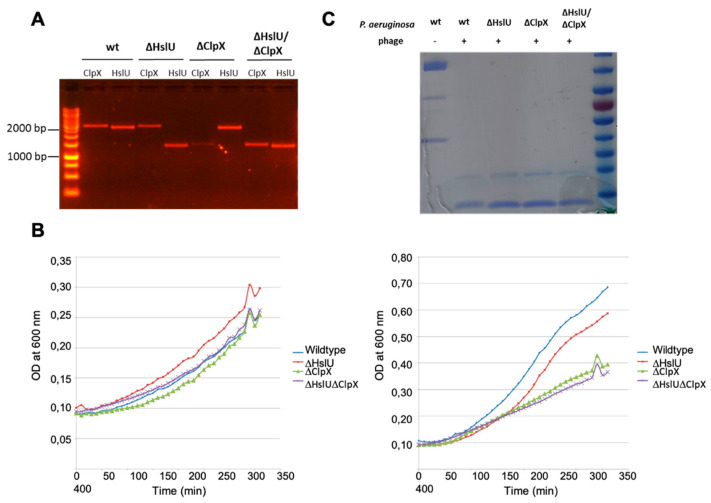
Analysis of *P. aeruginosa* knockouts of identified unfoldases. (**A**) Markerless knockout mutants created in genetic background of *P. aeruginosa rpoA::strep rac*. Shown are PCRs with primers 600 bp up- and downstream target gene. (**B**) Microbiological characterization of single and double knockouts, with (left) and without (right) induction of the phage GNAT. (**C**) Affinity purifications of RNAP complex using the Strep-fusion at the C-terminus of the α RNAP subunit, with and without phage infection.

Major bacterial proteases like ClpP and HslV associate with the ClpX and HslU ATPases, which recognize the correct protein substrates, unfold them, and ultimately translocate the denatured polypeptide to the protease for irreversible proteolysis [41,42]. These ATP-dependent proteases remove damaged, denatured and aberrantly folded proteins from the cell [43]. However, it is still poorly understood how these unfoldases discriminate between potential target proteins [44,45,46,47,48]. As our data suggested that Rac acetylates and influences the substrate specificity of these two unfoldases, we constructed single and double knockout *P. aeruginosa* strains of *clpX* and *hslU*. Although these mutants were viable, they displayed a clear reduction in fitness. Unfortunately, in all three mutants, the α RNAP subunit was still cleaved upon phage infection (Appendix A, Figure A7), suggesting redundancy in the proteases involved in this transcriptional shutdown.

## 4. Discussion

### 4.1. A Novel Mode of Phage-Induced Transcriptional Shutdown

Immediately following the injection of its genome, the obligatory lytic *Pseudomonas* phage phiKMV recruits the host RNAP complex using strong σ^70^ housekeeping promoters. Upon transcription of its single-subunit vRNAP (Gp26), the phage has to suppress the bacterial RNAP activity to favor the shift towards the late transcription of its genome by the vRNAP [24]. Protein Gp2 of the related coliphage T7, whose vRNAP is encoded among the early genes, binds the host RNAP through a β’ subunit jaw domain and inhibits transcription by preventing RNAP–promoter DNA interactions [49,50]. While phage phiKMV encodes a functional homologue of Gp2 directly upstream of the vRNAP gene [25], we show here that the phage Rac protein (Gp28) also contributes to transcriptional shutdown by inducing proteolytic removal of the α CTD from host RNAP. Our results suggest that the cleavage itself is catalyzed by host proteases which are yet to be identified and are likely redundant.

To date, only two types of phage-induced modifications to the bacterial RNAP complex were described: covalent RNAP modifications such as phosphorylation or ADP-ribosylation and modifications through RNAP-binding proteins [7]. Some of these modifications specifically target and/or disable the function of the bacterial α CTD. For example, the binding of Gp67 of phage G1 to the σ^A^_4_ subunit of the *Staphylococcus aureus* RNAP prevents the binding of the α CTD to its target DNA [51], while Ogr from coliphage P2 directly binds the *E. coli* α subunit to activate late transcription of the phage [52]. The proteolytic removal of the α CTD from the RNAP complex during infection of phiKMV-like viruses represents a distinct third type of host transcription machinery modification for host transcriptional shutdown.

### 4.2. Consequences of α CTD Cleavage During Phage Infection

In bacteria, the interaction between the α CTD and UP elements activates transcription from rRNA promoters up to 50-fold [53,54], which account for approximately 60% of the total RNA synthesis in exponentially growing *E. coli* cells [55]. Therefore, a significant part of the observed decrease in transcriptional activity in the presence of the truncated RNAP complex (Figure 2C) can be attributed to a drop in rRNA transcription. As cell growth is known to be limited by rRNA synthesis [38], the cleavage of the α CTD is also the likely mechanism through which Rac inhibits cell growth. Another part of the observed transcriptional shutdown might be caused by disturbed sequence-nonspecific interactions between the α CTD and *P. aeruginosa* promoters [56,57], although these are less well studied.

As a result of the declining bacterial transcription, the nucleotide pool available for phage transcription and genome replication increases substantially. Phage translation should not be hindered, as processed rRNAs are stable in prokaryotic cells [58] and previously formed ribosomes will be unaffected during the time course of infection. The influence of α RNAP cleavage on the transcription of phiKMV-type phages also seems limited, as no UP elements are associated with their early σ^70^-like promoters [23,33].

### 4.3. Posttranslational Control Mechanisms by Bacterial Viruses

The data presented in this paper show that expression of phage-encoded Gcn5-related acetyltransferase Rac mediates the truncation of the bacterial RNAP complex, possibly through interaction with the bacterial ClpP/X and HslV/U protease complexes (Figure 3 and Appendix A, Figure A6). However, other proteases have to be involved as well since the RNAP cleavage could still be detected in a *P. aeruginosa* PAO1 Δ*hslU*Δ*clpX* strain. A change in the enzyme specificity of such proteases based on PTMs like acetylation is an interesting avenue to pursue, providing flexibility towards selectivity and activity. PTMs enable bacteria to rapidly adapt to changing environments: instead of spending energy and time on gene transcription and protein translation, cells can quickly modify an existing protein to alter its activity from an inactive to an active state (or vice versa) in response to environmental changes [59]. Indeed, neutralization of the positive charge in proteases through acetylation might quickly enhance the recognition of the positively charged amino acids surrounding the cleavage site (EEQ/EDE) within the α RNAP linker. Lysine acetylation is indeed recognized as a relevant regulatory mechanism of gene transcription, because acetylated lysine residues are generally localized in DNA-binding domains and preclude the interaction of transcription factors with their DNA [17].

This unique system of viral transcriptional shutdown evolved within a *Pseudomonas* phage might point at the role of PTMs in protein degradation. Only a single example of this has been described, to our knowledge, by Trentini and colleagues [60]. They showed that *Bacillus subtilis* proteins phosphorylated on arginine residues by the McsB kinase are selectively targeted to the ClpC–ClpP proteolytic complex. Moreover, they proved that this arginine phosphorylation is required and sufficient for the degradation of substrate proteins [60].

In bacteria, acetylation has been shown to affect protein function and stability [20,61,62]. Although it might not be surprising that bacteriophages have evolved to adapt this common PTM to their favor, its study is still extremely limited. There are only a few documented examples of phage-induced PTMs, such as the ADP-ribosylating Alt protein of T4 and kinase Gp0.7 of phage T7, which target approximately 27 and 70 bacterial proteins, respectively [63,64]. Alawneh et al. [65] showed that one of the proteins ribosylated by T4 Alt is the *E. coli* MazF toxin, part of a Type III toxin–antitoxin system, thereby significantly decreasing its activity in protecting the bacterium from phage infection. To our knowledge, there is only one example of acetylation by phages. Recently, Dong et al. [66] showed that anti-CRISPR protein AvrVA5, identified on a prophage region in *Moraxella bovoculi*, is an acetyltransferase that acetylates the Lys635 residue of the host Cas12a protein (a type V CRISPR–Cas system), resulting in the complete loss of double-stranded DNA cleavage activity by steric hindrance [66]. Given the sheer pervasiveness of lysine acetylation of bacterial enzymes [16], the ever-increasing sensitivity of proteomic methods and the abundance of kinases and acetyltransferases in phage genomes, novel examples of phage-induced PTMs could be discovered in the near future.

## Appendix A

**Table A1:** Conservation of Rac among *Krylovirinae*.

Hit	Query Cover	*e*-Value	Percent Identity	Accession
hypothetical protein PPLUZ19_gp28 (Pseudomonas phage LUZ19)	100%	4.00 × 10^−120^	100.00%	YP_001671973.1
hypothetical protein PT2_gp33 (Pseudomonas phage PT2)	100%	2.00 × 10^−119^	100.00%	YP_002117812.1
putative acetyltransferase (Pseudomonas phage vB_PaeP_PE3)	100%	4.00 × 10^−119^	99.36%	QHZ59694.1
putative diguanylate cyclase/phosphodiesterase (Pseudomonas phage vB_PaeP_PAO1_Ab05)	100%	1.00 × 10^−118^	98.73%	YP_009125731.1
putative acetyltransferase (Pseudomonas phage vB_Pae-TbilisiM32)	100%	2.00 × 10^−118^	99.36%	YP_006299952.1
hypothetical protein MPK7_32 (Pseudomonas phage MPK7)	100%	2.00 × 10^−118^	98.09%	YP_008431340.1
putative diguanylate cyclase/phosphodiesterase (Pseudomonas phage vB_PaeP_130_113)	100%	9.00 × 10^−117^	96.82%	AVX47641.1
hypothetical protein (Pseudomonas phage DL62)	100%	1.00 × 10^−115^	96.18%	YP_009201889.1
hypothetical protein phiKMVp28 (Pseudomonas phage phiKMV)	100%	2.00 × 10^−113^	92.99%	NP_877467.1
hypothetical protein SPCB_034 (Pseudomonas phage vB_PaeP_SPCB)	100%	2.00 × 10^−112^	92.36%	QGJ86814.1
hypothetical protein PPphikF77_gp32 (Pseudomonas phage phikF77)	100%	2.00 × 10^−111^	91.08%	YP_002727851.1
hypothetical protein PPLKD16_gp28 (Pseudomonas phage LKD16)	100%	8.00 × 10^−111^	90.45%	YP_001522820.1
hypothetical protein AV952_gp40 (Pseudomonas phage LKA1)	73%	1.00 × 10^−21^	37.39%	YP_001522880.1
hypothetical protein SBWP25_0025 (Pseudomonas phage phi-2)	68%	6.00 × 10^−15^	31.78%	YP_003345490.1
hypothetical protein LIMEzero_ORF36 (Pantoea phage LIMEzero)	65%	3.00 × 10^−10^	35.92%	YP_004539109.1

**Table A2:** Result of the in silico analysis of the primary *Rac* sequence using various algorithms.

Algorithm	Identification	Probability (%)	*e*-Value
PHYRE	Acyl-CoA *N*-acyltransferase (Nat)	99.94	0.22
HHPRED	Acetyltransferase (GNAT)	90	1.8 × 10^−24^
PFAM	Acetyltransferase 7	NA	0.0029
